# Role of vascular endothelial dysfunction and metabolic eprogramming of immune cells in the development and ogression of atherosclerosis

**DOI:** 10.3389/fphys.2025.1624225

**Published:** 2025-09-03

**Authors:** Guo Zhili, Guan Zhimin, Zhang Nenghua

**Affiliations:** Jiaxing Hospital of Traditional Chinese Medicine, Jiaxing University, Jiaxing, Zhejiang, China

**Keywords:** metabolic reprogramming, glycolysis, oxidative phosphorylation, macrophages, T lymphocytes, endothelial cell dysfunction, atherosclerosis

## Abstract

The pathogenesis of atherosclerosis (AS) involves a complex interaction between vascular endothelial dysfunction and immunometabolic disorders. During the process of AS, vascular endothelial cells (ECs) are affected by multiple environmental stimuli (oxidative stress, shear force abnormalities) and undergo endothelial dysfunction, which is mainly manifested by a shift in energy metabolism toward aerobic glycolysis (the Warburg effect) and proliferation of ECs, which in turn leads to vascular remodeling and luminal narrowing. Meanwhile, infiltrating immune cellsundergo phenotypic polarization and functional alterations in response to stimulation of the AS microenvironment (hypoxia, inflammatory factor enrichment) and adapt to the energy demand through metabolic reprogramming (enhanced glycolysis, imbalance of fatty acid oxidation, FAO) to maintain their activation, proliferation, and inflammatory effects. However, such adaptive metabolic changes may exacerbate lipid phagocytosis and inflammatory responses, further promoting AS progression. Currently, key controversies remain in the therapeutic strategy of AS: should the therapeutic target of AS be centered oncorrecting vascular endothelial dysfunction or targeting the modulation of immune cell metabolic reprogramming? In addition, the causal relationship between the two has not been fully elucidated - is it endothelial dysfunction that triggers immune metabolic disorders, or is it the aberrant activation of immune cells that exacerbates endothelial damage? How do the two synergize to drive the cascade response in AS? In this article, the dynamic interplay between vascular endothelial dysfunction and immune-metabolic reprogramming in the development of AS will be systematically described and analyzed from the perspectives of molecular mechanisms and therapeutic targets, and case studies will be presented.

## 1 Introduction

The pathogenesis of AS is the result of the synergistic effect of vascular endothelial dysfunction and lipid metabolism disorders (Li et al., 2024). Abnormal deposition of lipids such as low-density lipoprotein (LDL) within the arterial wall activates the innate immune system and induces the migration of inflammatory leukocytes, such as monocytes and neutrophils, to the vascular intima (Roy et al., 2022). This physiological defense response initially helps to remove excess lipids, but persistent lipid accumulation triggers a chronic inflammatory cascade, leading to aberrant activation and aggregation of immune cells in the plaque microenvironment (Borén et al., 2020), which in turn creates a positive feedback loop of ‘inflammation-endothelial damage’ (Goldstein and Brown, 2015). Notably, the plaque microenvironment is characterized by hypoxic, acidic, and nutrient-limited conditions, which force infiltrating immune cells to adapt their energy utilization through metabolic reprogramming to meet the metabolic demands of proliferation, activation, and immune response. This adaptive metabolic alteration has become a non-negligible regulatory mechanism in the pathological process of AS. Additionally vascular endothelial inflammation is a host defensive tissueresponse to injury (Batista-Gonzalez et al., 2020). While acute inflammation contributes to pathogen clearance and promotes tissue repair, chronic inflammation or autoimmune dysregulation may be acentral driver of multiple diseases.

In vascular inflammation, ECs play a key role by regulating immune cell transport: immune cells are recruited to the site of injury through the dilated vascular systemand accumulate near the luminal edge in areas of the vasculature with low shear stress (e.g., arterial branches or bends) (Moore et al., 2018). Subsequently, immune cells undergo steps such as rolling, firm adhesion, and transendothelial migration to form close interactions with ECs and work together to orchestrate the inflammatory response within the vasculature (van Gils et al., 2012). Although existing studies have delved into the mechanisms of immune cell-ECs interactions, the following key questions remain unclear: 1) How does metabolic reprogramming of immune cells affect ECs dysfunction? 2) How do the two synergize during AS? 3) Does ECs dysfunction occur first or is metabolic reprogramming of immune cells triggered earlier at the onset of AS? At present, there is still a lack of endothelial cell and immune cell-specific conditional knockout models to study the bidirectionality and complexity of the interaction between endothelial dysfunction and immune metabolic reprogramming in AS, which limits the research on understanding the ‘initiation’ sequence. The aim of this paper is try to analyze the above questions with a view to providing new targets for the treatment of cardiovascular and cerebrovascular diseases.

## 2 Mechanisms of vascular endothelial dysfunction

**FIGURE 1:**
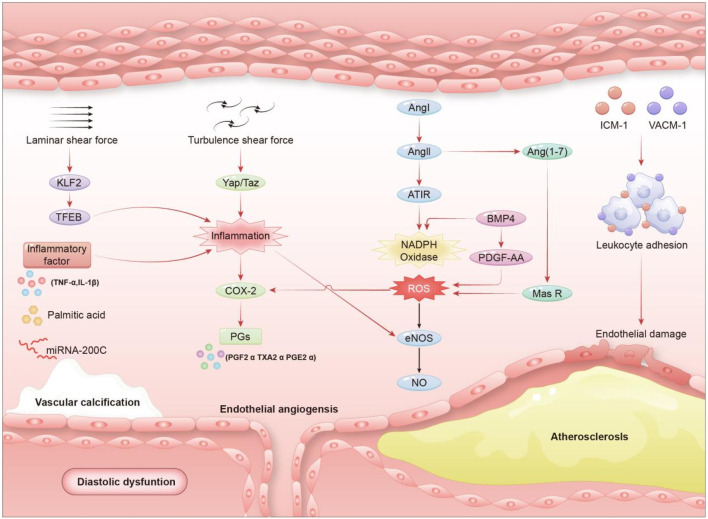
Illustrates a cascade reaction initiated by metabolic disorders, progressing through chronic inflammation, and ultimately leading to abnormal vascular structure and function. This schematic provides valuable insights into the underlying mechanisms and potential therapeutic targets for related diseases.Inflammatory response: Palmitic acid (PA) and inflammatory mediators such as TNF-α and IL-19 contribute to the activation of inflammatory pathways by stimulating TFEB and Yap/Taz signaling, which in turn enhances the expression of COX-2 and the production of prostaglandins (PGs). Oxidative stress: Reactive oxygen species (ROS) generated by NADPH oxidase, together with factors including BMP4 and PDGF-AA, play a synergistic role in inducing endothelial dysfunction and vascular calcification. An imbalance between Ang1 (angiopoietin 1) and AT1R (angiotensin receptor 1), along with dysregulation of the Ang (1–7)/MasR pathway, further aggravates vascular damage, eventually contributing to the development of atherosclerosis and diastolic dysfunction. Elevated expression of cell surface adhesion receptors, such as ICAM-1 and VCAM-1, promotes excessive adhesion of leukocytes to the vascular endothelium, triggering localized inflammation, impairing endothelial barrier integrity and vasodilatory capacity, thereby resulting in endothelial dysfunction. Core relationship chain: Adhesion receptor ↑ → Leukocyte adhesion ↑ → Inflammation ↑ → Endothelial injury/dysfunction → Vascular endothelial dysfunction.

Vascular endothelial dysfunction is not only characterized by disturbances in vasoconstrictive and diastolic function, but also encompasses enhanced endothelial inflammation, increased leukocyte adhesion, oxidative stress, enhanced endothelial proliferation and migration, and impaired endothelial barrier function (Zhang et al., 2022). Endothelial dysfunction is not only indicative of cardiovascular disease, but also an important driver of its ongoing progression. Oxidative stress and inflammatory responses are central components leading to vascular endothelial impairment and mediate multiple risk factors causing endothelial dysfunction. Vascular endothelial dysfunction is a complex pathophysiologic process regulated by multiple factors, and the mechanisms of endothelial dysfunction induced by different diseases or risk factors vary. Inflammation and oxidative stress are key components in causing endothelial dysfunction, and both jointly regulate endothelium-dependent vasoconstrictive and diastolic functions by affecting the expression and activity of COX2 and eNOS (Xue-Bin, 2020) (As shown in Figure 1).

## 3 Metabolic adaptation of vascular endothelial structures and its relation to AS

### 3.1 Metabolic adaptation of vascular smooth muscle cells in relation to AS

Metabolic adaptation of vascular smooth muscle cells (VSMCs) serves as a central component of atherogenesis and development of AS, and their metabolic reprogramming drives phenotypic transitions, inflammatory responses, and plaque dynamics evolution by altering energy metabolic pathways. In AS, the transition of SMC from contractile (differentiated state) to synthetic (de-differentiated state) is characterized by the weakening of mitochondrial oxidative phosphorylation (OXPHOS) and the shift to rely on glycolytic pathways for rapid energy supply, which further triggers a series of metabolic imbalances: under inflammatory microenvironmental stimuli, upregulation of CD36 expression on the surface of SMC promotes abnormal uptake of oxidized low-density lipoprotein (oxLDL). uptake, leading to intracellular lipid accumulation and foamy degeneration; at the same time, inhibition of carnitine palmitoyltransferase 1 (CPT1) activity causes fatty acid β-oxidation impairment, exacerbating lipotoxicity; and impaired function of the mitochondrial electron transport chain (ETC) not only induces excessive accumulation of ROS, which can lead to senescence, apoptosis, or autophagy defects of SMC, but also damage to mitochondrial DNA through the concomitant oxidative stress. mitochondrial DNA, forming a vicious cycle of energy metabolism disorders (Wu et al., 2022).

Under physiological conditions, the differentiation of the media membrane VSMCs exhibits a contractile phenotype that regulates vascular diameter and blood flow. However, in response to injury, VSMCs transform their phenotypes into synthetic phenotypes dominated by migration and proliferation activities (Falk, 2006). In AS, VSMCs from the media migrate to the intima in response to growth factors produced by foam cells (VSMCs or macrophage-derived) or intimal ECs. In addition, IL-1 produced by macrophages can enhance the production of endogenous PDGF in VSMCs. Once it enters the intima, it will autocrine and lead to its proliferation. VSMCs with synthetic phenotypes will also increase the production of ECM components, such as interstitial collagen, elastin and proteoglycans. These proliferating VSMCs, along with the production of ECMs, generate a fibrous cap that covers the atherosclerotic plaque that is forming, thereby enveloping the lesion and preventing it from rupturing. If the production of mitogens does not cease, VSMCs will not switch back to the contractile phenotype, which is conducive to the development of the lesion. The characteristics of the fibrous cap, such as thickness, cellular structure, matrix composition and collagen content, are important determinants of plaque stability (Chen et al., 2022).

### 3.2 Metabolic adaptation of vascular ECs in relation to AS

ECs act as a selective barrier between the blood and underlying tissues, controlling oxygen and nutrient supply and participating in immune surveillance. Under physiological conditions, ECs maximize energy production through anaerobic glycolysis, which accounts for nearly 85% of ATP production compared with oxidation of glucose, fatty acids, or amino acids through the electron transport chain (Bierhansl et al., 2017). This metabolic pattern is also influenced by hemodynamics, which together shape the morphology and function of ECs. For arterial ECs, activities including regulation of vascular tone and redox homeostasis and control of hemostasis, thrombosis, and inflammatory responses are achieved by coordinating the biosynthesis and degradation of vasoactive mediators, extracellular matrix components, growth factors, cytokines, and hormone-like molecules, prostaglandins and autocrine hormones, and the transport and metabolism of lipoproteins (Cochain et al., 2018). These multiple mechanisms involved in tissue homeostasis largely explain why dysfunction of ECs is a critical step in the development of AS.

### 3.3 Impact of systemic metabolic changes on endothelial barrier integrity and function

Altered shear stress and enhanced intra-arterial curvature and turbulent blood flow at branch points increase the opportunity for ECs to trap LDL and other cholesterol-rich apoB particles into the vascular intima via cytotrophy, a process triggered by enhanced endothelial permeability, which, in combination with hypercholesterolemia, is thought to be a key initiating point for driving the transformation of ECs into a pro-inflammatory, pro-thrombotic, and atherogenic layer. Lipoprotein-carried lipids (mainly in oxidized form) induce a strong activation cascade of inflammatory responses through activation of endothelial NF-kB; this event leads to the production of proinflammatory cytokines (TNFα, IL1β), chemotactic proteins (CCL1, CX3CL1, CCL5), and leukocyte adhesion molecules (E-selectin and VCAM1), which are critical for immune cell recruitment in the vessel wall (King et al., 2017).

ECs readily bind to triglyceride-rich lipoproteins (TGRLs), which can then promote the expression of adhesion molecules (VACM1, E-selectin, and CD31) and pro-inflammatory genes (PAI1, CCL2, and IL6). This expression is reflected more clearly in patients with hypertriglyceridemia or elevated levels of small, dense atherosclerotic low-density lipoprotein (sdLDL). Studies have shown that removal of cholesterol from ECs, via high-density lipoprotein (HDL), exerts anti-inflammatory, antithrombotic, and antiprotein hydrolyzing activities on ECs with the potential to counteract the proatherosclerotic potential induced by lipid accumulation (Domanski et al., 2020). Endothelial function is closely related to its metabolism under physiologic conditions as well as when metabolic capacity is exceeded (e.g., in the presence of excess extracellular nutrients) (Perrotta, 2022). In the latter case, ECs may undergo specific adaptations that affect the maintenance of barrier integrity and vascular tone and, in the long run, also contribute to AS by affecting the function of other cells present in the arteries (e.g., vascular smooth muscle cells) and/or promoting immunity.

### 3.4 Hemodynamic and endothelial dysfunction

Hemodynamic forces constitute local risk factors for the formation of AS, as they promote endothelial dysfunction. Vulnerable areas are mainly located in regions where laminar flow is disturbed due to flow separation, recirculation or reattachment. This turbulence generates temporal and spatial gradients, thereby leading to a higher oscillation index and lower shear stress. In addition, turbulence is also conducive to the infiltration of lipoproteins into the intima of blood vessels. Firstly, LDLwill remain in these areas for a longer time. Secondly, turbulence can cause physical damage to the integrity of the endothelium, thereby promoting lipoprotein infiltration. Furthermore, another fundamental connection between hemodynamics and AS formation depends on the expression of multiple endothelial genes regulated by mechanical blood stimulation. These genes, such as eNOS or platelet adhesion molecule 1 (PECAM-1), have shear stress response elements (SSRE) in their promoters, which contribute to plaque formation. Meanwhile, prolonged exposure of EC to undisturbed laminar flow promotes the upregulation of endothelial nitric oxide synthase (eNOS), thereby increasing their nitric oxide (NO) synthesis. The different molecular responses of ECs depend on the blood flow pattern, highlighting the role of hemodynamic forces in endothelial dysfunction (Falk, 2006).

## 4 Altered metabolic and functional characteristics of different immune cells in AS and the relationship with the development of AS

**FIGURE 2:**
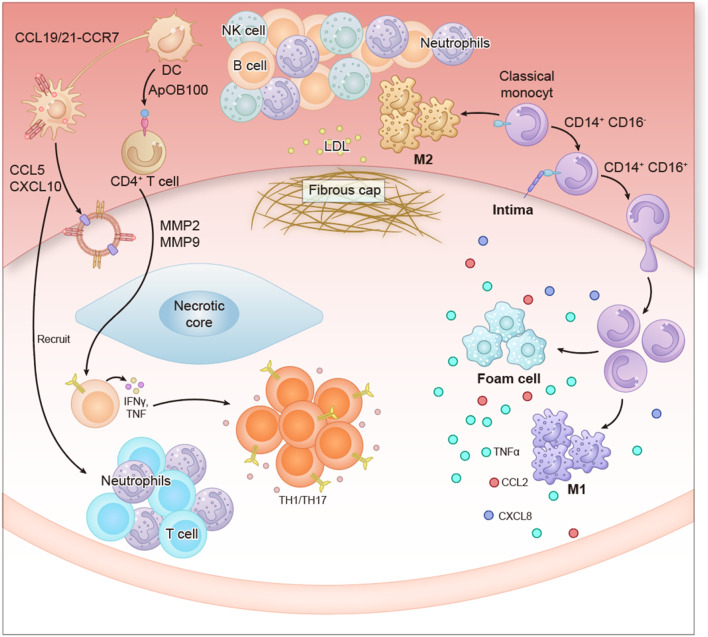
Shows the complex interaction network between immune cells and inflammatory factors during the development of Atherosclerosis.

Imbalance in immune homeostasis is a key factor driving the development of AS.The pathologic process of AS essentially reflects a dysfunctional dynamic balance between pro- and anti-inflammatory immune cells. In intrinsic immune regulation, monocytes, macrophages, T-lymphocytes, and dendritic cells play a central role, while B-lymphocytes and natural killer cells (NK cells) are involved in partial regulation. Macrophages play a crucial role in AS progression through their unique metabolic and functional reprogramming mechanisms (He et al., 2022) (Figure 2).

It mainly involves the following key processes: Recruitment and activation of immune cells: The chemokine CCL19/21-CCR7 signaling promotes the migration and antigen presentation of dendritic cells (DCS). Neutrophils, B cells, NK cells and CD4^+^ T cells are recruited to the lesion site by chemokines such as CCL5 and CXCL10, and release pro-inflammatory factors (such as IFN-γ and TNF-α). Monocytes (CD14++ CD16^−^classical subpopulation) differentiate into macrophages and further form Foam cells, which secrete TNF-α, CCL2 and CXCL8, intensifying the inflammatory response. Plaque instability and Fibrous cap degradation: MMP2 and MMP9 (matrix metalloproteinases) are activated by immune cells to degrade the fibrous cap, leading to plaque rupture and triggering acute cardiovascular events. The participation of Th17 cells further promotes the formation of the inflammatory microenvironment. Pathological outcome: Persistent inflammatory response and extracellular matrix destruction jointly promote the progression of atherosclerotic plaques, which may eventually lead to clinical consequences such as vascular stenosis and thrombosis. The content in the figure integrates the synergistic effects of innate and adaptive immunity, highlighting the core role of inflammation in atherosclerosis and providing a theoretical basis for related therapeutic targets (such as chemokines and MMPs).

### 4.1 Monocytes

During the formation and progression of atherosclerotic plaques, monocytes are involved in the inflammatory response and plaque progression through a variety of mechanisms, and their function, phenotype, and recruitment patterns are significantly altered. Monocytes and plaque-resident macrophages have different metabolic pathways. Monocytes and their bone marrow progenitors are exposed to a variety of stimuli, including lipoproteins, glucose, and dietary/microbial sources, which can alter the monocyte phenotype by altering its phenotype. Once inside the atherosclerotic plaque, macrophage metabolism can be further altered by stimuli in the atherosclerotic plaque microenvironment, including hypoxia, modified lipoproteins, and cytokines. Macrophage activation also alters lipid metabolic patterns. For example, IL4-activated mouse macrophages increased fatty acid uptake and enhanced FAO, whereas fatty acid uptake was reduced and FAO was inhibited in macrophages activated by co-stimulation with LPS (lipopolysaccharide) and IFNγ (gamma interferon).

#### 4.1.1 Monocyte recruitment and infiltration

Chemokine-mediated recruitment of immune cells plays a key role in atherosclerotic plaque formation: on the one hand, the chemokine CCL2 (MCP1)secreted by vascular ECs and plaque ECs drives monocytes to migrate directionally from the circulatory system to the vascular wall by binding to the CCR2 receptor on the surface of the monocytes, and on the other hand, the plaque microenvironment CX3CL1 (Fractalkine), promotes stableadhesion of monocytes to ECs via CX3CR1 receptors and further directs their transendothelial migration to the subendothelial region. In addition, under the stimulation of oxLDL and inflammatory factors (e.g., TNFα, IL1β), activated ECs overexpress the adhesion moleculesVCAM1 and ICAM1, which further intensify the process of immune cell capture and retention by enhancing monocyte-endothelial interactions (Wu et al., 2023), and these synergistic recruitment mechanisms work together to promote inflammatory cell accumulation in the vascular wall, and these synergistic recruitment mechanisms together promote the sustained accumulation of inflammatory cells in the vascular wall.

#### 4.1.2 Differentiation of monocytes into macrophages with foam cell formation

After monocyte entry into the plaque, monocytes are stimulated by pro-inflammatory factors (e.g., IFNγ, GM-CSF) to differentiate mainly into M1-type macrophages, a pro-inflammatory phenotype that forms typical foam cells by phagocytosis of large amounts of ox-LDL, and, in the microenvironment dominated by anti-inflammatory factors (e.g., IL-4, IL-13),they may differentiate into M2-type with repair function macrophages, the latter playing a plaque-stabilizing role by inhibiting the inflammatory response and promoting fibrous cap formation (Mitra et al., 2011). This differentiation process is accompanied by significant metabolic reprogramming features: in the pro-inflammatory polarized state, enhanced glycolysis driven by the HIF1α/mTOR signaling pathway provides energetic support for inflammatory factor secretion; at the same time, the impaired cholesterol efflux due to downregulation of cholesterol transporter protein (ABCA1/ABCG1) expression in conjunction with lipid metabolism imbalance aggravates the intracellular lipid accumulation, which ultimately promotes macrophage transformation to foam cells (Lin et al., 2016).

#### 4.1.3 Monocytes and plaque instability

Monocyte-derived macrophages weaken the fibrous cap structure by secreting matrix metalloproteinases (MMPs) to degrade collagen fibers, while releasing pro-inflammatory factors such as IL-6 and IL-12 to activate T cells and ECs, forming an inflammatory cascade amplification effect; on the other hand, lipids and inflammatory mediators released by apoptosis of foam cells continue to enlarge the necrotic core, which is further exacerbated by persistent mononuclear cell infiltration This pathologic process is further exacerbated by the continuous monocyte infiltration. To address these key mechanisms, current intervention strategies focus on three levels: promoting M2-type anti-inflammatory polarization through PPARγ agonists or IL-10 delivery to enhance plaque stability; blocking foam cell formation by inhibiting glycolysis with 2-deoxyglucose (2DG) or enhancing cholesterol efflux with LXR agonists (Rosenson et al., 2012); and targeting the removal of pro-inflammatory monocytes with nanoparticles. Notably, classical monocytes in atherosclerotic plaques primarily drive early inflammation and necrotic core formation, whereas nonclassical monocytes may play a role in late repair, and this subtype-specific functional difference provides a theoretical basis for precise intervention. Targeted modulation based on monocyte recruitment, polarization state, and metabolic reprogramming is emerging as a novel therapeutic direction for stabilizing vulnerable plaques and delaying disease progression.

### 4.2 Dendritic cells (DCs)

As specialized antigen-presenting cells, play a key immunomodulatory role in AS. Under physiological conditions, DCs are widely distributed in healthy arterial tissues; in AS lesions, DCs recognize and uptake “atherosclerosis-associated antigens”, such as oxLDL, and subsequently migrate to lymphoid organs and vascular epithelium through the CCL19/21-CCR7 chemokine axis to participate in the activation and regulation of lymphocytes (Sprague et al., 2014). The immune functions of DCs are finely regulated by Cholesterol metabolism is finely regulated.Excess lipid accumulation in AS lesions activates NLRP3 inflammasome, which in turn enhances the antigen-presenting capacity of DCs and promotes lymphocyte activation. Notably, lymphocyte activation was accompanied by substantial metabolic reprogramming, characterized by a shift from lipid oxidation metabolism in the resting state to a glycolysis-dependent metabolic profile in the activated state.Similarly, DCs activation showed significant lipid metabolic reprogramming, and these metabolic changes profoundly affected the immune function of DCs. DCs exhibits dual immunomodulatory characteristics. It achieves pro-inflammatory effects by presenting antigens such as oxLDL and inducing the differentiation of pro-inflammatory T cell subsets like Th1 cells and the release of inflammatory mediators. Additionally, it exerts anti-inflammatory effects through mechanisms such as promoting the differentiation of Treg cells and the secretion of IL-10 (Daissormont et al., 2011).

#### 4.2.1 Impact of altered DC function on plaque progression

DCs presents the ApoB100 antigenic peptide in oxLDL to CD4^+^T cells, driving the expansion of Th1 and Th17 clones (such as secreting IFNγ and IL17). DCs secretes pro-inflammatory factors such as IL12, IL6, and IL23, promoting the differentiation of Th1/Th17. Pro-inflammatory factors directly destroy the endothelial barrier and recruit more monocytes and neutrophils. At the same time, DCs releases chemokines such as CCL5 and CXCL10, recruiting T cells and monocytes into plaques. Activated DCs inhibit the differentiation of regulatory T cells by reducing the secretion of TGFβ and retinoic acid (RA). DCs secretes matrix metalloproteinases (MMP2, MMP9), which degrade the collagen in the fibrous cap of plaques, increase the risk of rupture, induce apoptosis of smooth muscle cells through the FasL or TRAIL pathways, and weaken the plaque repair ability (Grao-Cruces et al., 2022).

However, it remains unclear whether DCs are directly involved in the initiation of atherosclerotic plaques (e.g., during endothelial injury) or if their role is limited to amplifying inflammation at later stages. The specific functional roles of distinct DCs subpopulations, such as conventional dendritic cells type 1 (cDC1) and type 2 (cDC2), within plaques have yet to be fully elucidated. In atherosclerotic plaques, DCs undergo significant alterations, including increased numbers, enhanced activation phenotype, lipid accumulation, and subpopulation imbalance. These alterations lead to dysfunctional DCs antigen presentation, overproduction of proinflammatory factors, and disruption of immune tolerance, ultimately exacerbating Th1/Th17-driven inflammatory responses and plaque instability. Future studies are needed to further analyze the DCs subpopulation-specific mechanisms and explore DCs-targeted immunomodulatory strategies to stabilize plaques.

### 4.3 T-lymphocytes

T lymphocytes play a core regulatory role in AS through metabolic reprogramming and functional polarization: In the resting state, T cells mainly rely on FAO, OXPHOS, and glutamine metabolism to maintain energy homeostasis. However, after antigen stimulation (such as the recognition of endogenous antigens like oxLDL within plaques), T cell metabolism rapidly shifts to be dominated by glycolysis (Warburg effect), significantly enhancing the intensity of glycolysis and inhibiting FAO to meet the requirements of rapid proliferation and effeffect functions (Chajadine et al., 2024). This metabolic adaptability drives T cell activation. Clinical studies have shown that the number of T cells in unstable coronary plaques increases and there is specific clonal expansion (such as CD4^+^ T cells targeting apoB100), in contrast to the absence of such clonal expansion in peripheral blood, confirming that antigen-driven T cells preferentially recruit to the plaque area. Different T cell subsets regulate the disease process by secreting specific cytokines: pro-inflammatory Th1 cells secrete IFN-γ to activate macrophages and inhibit collagen synthesis, leading to thinning of the fibrous cap; Th17 cells recruit neutrophils through IL-17 to aggravate vascular inflammation. Regulatory T cells (Treg) inhibit the Th1/Th17 response by secreting IL-10 and TGF-β, maintain immune tolerance and delay plaque progression. CD8^+^ T cells exhibit a dual effect - their cytotoxicity can eliminate diseased cells, but when overactivated, they release IFN-γ, exacerbating endothelial injury (Joly et al., 2018). This imbalance of the metabolism-functional axis is directly related to plaque stability, suggesting that targeting T-cell metabolic reprogramming (such as inhibiting glycolysis) may be a new strategy for intervening in AS.

#### 4.3.1 T cells in atherosclerotic plaques increase fatty acid uptake

The metabolic mode of T cells is closely related to their functional state: effector T cells (e.g., Th1, Th17), which usually rely on glycolysis for rapid energy supply, support the synthesis and secretion of inflammatory factors (e.g., IFNγ, IL17). Memory T cells or regulatory T cells: tend to maintain long-term survival and function through OXPHOS and FAO. Characterization of T-cell metabolism in the plaque microenvironment High-lipid environment drives fatty acid uptake: high levels of oxLDL and free fatty acids are present in atherosclerotic plaques (Link et al., 2006). Local T cells in plaques (especially pro-inflammatory subtypes such as Th1 and Th17) may enhance fatty acid uptake through CD36 receptors for energy supply and membrane synthesis. Studies have shown that a high-fat diet induces high expression of T-cell fatty acid transporters (such as CD36 and FATP1) in atherosclerotic plaques in mouse models (Siddiqi et al., 2015). Th17 cells maintain their pro-inflammatory function in a lipid-enriched environment by enhancing FAO.

However, the dependence on fatty acid metabolism may be different for different T cell subsets (e.g., Th17 depends on FAO, whereas Th1 is more dependent on glycolysis). T cell metabolic profiles within plaques may change dynamically with disease stage (Thomas et al., 2021). However targeting T cell lipid metabolism (e.g., inhibiting CD36 or PPARγ) may reduce pro-inflammatory T cell infiltration. Enhancement of Treg function (e.g., IL2 therapy) or specific clearance of pathogenic clonal T cells may stabilize plaques. In atherosclerotic plaques, T cells (especially Th1 and Th17 subtypes) adapt to the high-fat microenvironment by enhancing fatty acid uptake, supporting their activation and proinflammatory function. The high-fat environment not only increases the number of T cells within the plaque, but also drives specific clonal expansion via lipid antigens, exacerbating immune imbalance and plaque instability. Targeting T cell metabolism or clonal expansion may become a new direction for future therapy (Dai et al., 2022).

### 4.4 Macrophages

The initiation of AS stems from the subendothelial retention and accumulation of apolipoprotein B (apoB)-containing lipoproteins (Shea et al., 2019), and macrophages have long been regarded as the central drivers of its progression: infiltration of circulating monocytes and proliferation of locally resident macrophages make them the most abundant population of immune cells in the plaque, and these cells bind to the activated endothelium via adhesion molecules. These cells bind to the activated endothelium via adhesion molecules and migrate to the intimal layer, where they uptake oxLDL and convert it into foam cells, directly contributing to the formation of early fatty streaks; at the same time, macrophages secrete proinflammatory cytokines (TNF-α, IL-1β) and chemokines (CCL2, CXCL8) in response to stimulation of the atherosclerotic microenvironment (e.g., proteoglycan and other danger signals), recruiting more leukocytes and amplifying the inflammatory cascade response. In addition, macrophages profoundly influence plaque vulnerability by bi-directional regulation of extracellular matrix metabolism - both secreting collagen to enhance fibrous cap stability and releasing matrix metalloproteinases (MMPs) to degrade the matrix (Peng et al., 2012). Recent studies have further revealed the critical role of metabolic reprogramming of monocytes/macrophages (e.g., enhanced glycolysis, inhibition of cholesterol efflux), in the progression of the disease: macrophages in early plaques efficiently phagocytose modified lipoproteins to form foam cells via receptors such as CD36, whereas lipid overload and persistent inflammation in the core region of late plaques lead to apoptotic necrosis, release of pro-inflammatory contents, and formation of anecrotic core in the macrophage. This ultimately drives the transformation of the plaque from a stable to an unstable state.

#### 4.4.1 Macrophage types in atherosclerotic plaques

Foam cells predominate in Plaque microenvironment: hyperlipidemic features of the plaque microenvironment lead to oxidation of LDL to form oxLDL, which is phagocytosed by macrophages to form foam cells. Inflammatory signals: IFNγ, TNFα, *etc.*, drive M1 polarization. Hypoxia and metabolic stress: hypoxia in the plaque core promotes glycolysis.

Plaques are dominated by M1-type macrophages: plaques highly express M1 markers such as iNOS and IL6. pro-inflammatory factors (e.g., IL1β) secreted by M1 cells exacerbate endothelial injury and plaque rupture risk. In animal models, inhibition of M1 polarization reduces plaque size. Mechanism of action: Foam cell formation: M1 macrophages take up large amounts of oxLDL but have impaired cholesterol efflux capacity (such as ABCA1/ABCG1), leading to lipid accumulation. Matrix degradation: secretion of matrix metalloproteinases, such as MMP9, degrades fibrous cap collagen and triggers plaque rupture. Necrotic core expansion: pro-inflammatory signaling induces apoptosis and necrotic material further recruits inflammatory cells .The plaque is mainly composed of M1-type macrophages: M1 markers such as iNOS and IL6 are highly expressed in the plaque. Pro-inflammatory factors secreted by M1 cells (such as IL1β) increase the risk of endothelial injury and plaque rupture. In animal models, inhibiting M1 polarization can reduce plaque area. M1-type macrophages consume large amounts of oxLDL, but their cholesterol efflorescation capacity is impaired, leading to lipid accumulation. At the same time, they secrete matrix metalloproteinases such as MMP9, which degrade fibrous cap collagen, causing plaque rupture. Pro-inflammatory signals induce apoptosis, and necrotic substances further recruit inflammatory cells (Ming-Tai et al., 2020).

The contradictory role of M2-type macrophages: Potential protective effect, a few M2 cells inhibit inflammation by secreting IL10 and promote the stability of the fibrous cap. Promote collagen synthesis and enhance the structural integrity of plaques (Guo et al., 2018). The pro-inflammatory microenvironment within the plaque (such as oxLDL and IFNγ) inhibits M2 polarization. M2 cells may aggravate vascular wall hardening by promoting fibrosis. Macrophage metabolism is plastic. M1/M2 is not an absolute dichotomy and exists in intermediate states. Targeting metabolic pathways (such as inhibiting glycolysis or enhancing FAO) may become a new therapeutic strategy. The current strategy aims to reduce M1 polarization while enhancing M2 repair function. Future treatments may stabilize plaques and prevent cardiovascular events by regulating the metabolic phenotype of macrophages and balancing the inflammatory and repair processes (Do et al., 2013).

#### 4.4.2 The participation of other immune cells

B lymphocytes: The role of B cells is complex, and different subgroups have opposite functions. Pro-inflammatory B cells secrete pro-inflammatory antibodies (such as IgG), activate the complement system and promote the formation of foam cells. Regulatory B cells inhibit T cell activation by secreting IL10. B1 cells secrete natural IgM antibodies, promoting the clearance of apoptotic cells and inhibiting inflammation (Ketelhuth and Hansson, 2016).

Natural killer cells (NK cells): They directly kill diseased cells by secreting IFNγ and perforin, but excessive activation may exacerbate endothelial damage. Mast cells: Release mediators such as histamine and chymotrypsin, promoting increased vascular permeability and plaque rupture (Bonacina et al., 2021).

In conclusion, the vast majority of macrophages present in atherosclerotic plaques originate from circulating monocytes. Mononucleosis is an independent risk factor for cerebrovascular diseases, associated with increased AS and impaired inflammatory resolution. The interaction between dendritic cells and T/B lymphocytes dominates the adaptive immune response, while the release of inflammatory factors, dysregulation of antigen presentation and changes in the proportion of cell subsets are the key mechanisms for plaque formation and rupture. Macrophages play a crucial role in the progression of AS through their unique metabolic and functional reprogramming mechanisms. The following focuses on the role of macrophages in the occurrence and development of AS.

## 5 Lipid accumulation promotes anaerobic glycolysis but not lipid metabolism in monocyte macrophages

### 5.1 Lipid accumulation leads to mitochondrial function impairment

Excessive FFA and cholesterol crystallization can inhibit the activity of electron transport chain (ETC) complexes (such as complexes I and III), reduce the efficiency of OXPHOS, induce ROS bursts, and result in mitochondrial DNA damage and a decrease in membrane potential. Cholesterol esters accumulate in lysosomes (such as NPC1 deficiency), which hinders mitochondrial uptake of fatty acids and limits the supply of β -oxidation substrates. After mitochondrial function is impaired, cells cannot effectively utilize FAO for energy supply and are forced to turn to glycolysis to maintain ATP production (Tadokoro et al., 2020).

Inflammatory signaling drives glycolytic dominance:Lipid overload activates TLR4/NFκB and NLRP3 inflammasomes, inducing macrophages to polarize towards pro-inflammatory M1 type. M1 macrophages are characteristically dependent on glycolysis as they require rapid energy supply to support the synthesis and secretion of inflammatory factors such as TNFα and IL6 (Orecchioni et al., 2019). The lipid microenvironment stabilizes the synthesis of hypoxia-inducible factor (HIF1α) through ROS and inflammatory signaling. HIF1α upregulates glycolytic enzymes and inhibits the entry of pyruvate into mitochondria (inhibiting PDH through PDK1), forcing metabolism to flow towards glycolysis.

### 5.2 Molecular mechanisms of impaired lipid metabolism

FAO key enzymes are inhibited: lipid overload inhibits CPT1 activity, preventing long-chain fatty acids from entering the mitochondria for β-oxidation. Enhanced activity of acetyl coenzyme A carboxylase (ACC) promotes malonyl coenzyme A accumulation and further inhibits CPT1 (Yan et al., 2023). Accumulation of lipid droplets inhibits the autophagic lysosomal pathway, resulting in the inability to efficiently hydrolyze stored triglycerides (TGs) to free fatty acids for energy. Overexpression of lipid droplet surface proteins (e.g., PLIN2) prevents lipases (e.g., ATGL) from approaching lipid droplets, limiting lipolysis (Coliva et al., 2019).

Macrophages in atherosclerotic plaques highly express glycolytic enzymes and have significantly elevated mitochondrial ROS levels. Inhibition of glycolysis (e.g., 2DG treatment) reduces foam cell formation and inflammatory factor release. Therapeutic potential: targeting HIF1α or glycolytic enzymes may restore metabolic homeostasis and reduce inflammation. Enhancing mitochondrial function or promoting FAO (e.g., CPT1 agonists) may be new strategies. Lipid accumulation forces monocytes/macrophages to rely on anaerobic glycolysis for energy supply by impairing mitochondrial function, activating inflammatory signals and inhibiting FAO. This metabolic reprogramming not only meets the energy demands of cells under lipotoxic stress, but also exacerbates the inflammatory response and plaque progression, creating a self-reinforcing pathological cycle. Dissecting this mechanism provides new ideas for targeting metabolic modulation for the treatment of AS (Zhuang et al., 2021).

## 6 Typical cases of macrophages adjusting their immune function in response to inflammatory changes and the metabolic reprogramming that regulates the immune response

### 6.1 Bacterial infection (M1-type polarized) immune response

Secretion of pro-inflammatory factors (TNFα, IL6, IL1β), recruitment of neutrophils, direct killing of pathogens. Bacteria are cleared via ROS and NO-mediated oxidative bursts. Metabolic reprogramming: enhanced glycolysis: relies on the “Warburg effect” for rapid energy supply to support inflammatory factor synthesis. Activation of HIF1α by hypoxia or inflammatory signals upregulates glycolytic enzymes such as HK2. Promotes glycolysis and inhibits mitochondrial oxidative phosphorylation. Pentose phosphate pathway (PPP) activation: generates NADPH, maintains ROS production and antioxidant defense (Fang et al., 2022).

### 6.2 Parasite infection/tissue repair (M2 type polarization) immune response

Secretion of anti-inflammatory factors to promote tissue repair and fibrosis. Enhanced phagocytosis (efferocytosis) to remove apoptotic cells. Metabolic reprogramming: OXPHOS and fatty acid oxidation (FAO): active mitochondrial function, dependent on fatty acid and glutamine metabolism. Key regulatory molecules: AMPK: activates FAO and mitochondrial biosynthesis. PPARγ/δ: promotes fatty acid uptake and oxidation, inhibits inflammatory signaling. Enhanced urea cycle: catabolizes arginine via arginase 1 (Arg1), inhibits NO synthesis, and reduces tissue damage (Yin et al., 2023).

### 6.3 Tumor microenvironment (TAMs, tumor-associated macrophages)

Immunoreactivity: inhibits T cell activity, promotes angiogenesis and tumor metastasis (M2-like phenotype). Metabolic reprogramming: FAO and arginine metabolism: tumor cells release lactate and lipids, driving TAMs to rely on FAO for energy. Arginase 1 (Arg1) is highly expressed, depleting the microenvironment of arginine and suppressing T cell function. Key regulatory molecules: STAT6: IL4/IL13 signaling activates STAT6 and promotes M2 polarization. Lactate: enhances glycolysis and immunosuppressive phenotype through HIF1α and NFκB (Tharp et al., 2024).

### 6.4 Effects of smoking on macrophage metabolic reprogramming

Smoking disrupts macrophage metabolism and promotes inflammation and disease progression through multiple components (e.g., nicotine, tar, ROS): 1. Activation of pro-inflammatory phenotypes (M1 polarization) ROS and oxidative stress: free radicals in cigarette smoke directly injure mitochondria, inhibit OXPHOS, and force cells to rely on glycolysis. Activation of NLRP3 inflammatory vesicles promotes IL1β secretion.Enhanced HIF1α signaling: cyanide in cigarette smoke inhibits the mitochondrial respiratory chain, mimicking hypoxia, stabilizing HIF1α, and up-regulating glycolytic enzymes. 2. Inhibition of anti-inflammatory and reparative functions (impaired M2 polarization): mitochondrial dysfunction: nicotine inhibits AMPK activity, reducing FAO and mitochondrial biosynthesis. Reduces PPARγ expression and hinders M2-related gene transcription. Imbalance of arginine metabolism: smoke induces high expression of iNOS (M1 marker), increasing NO production, while inhibiting Arg1 (M2 marker), impeding repair. 3. Specific metabolic alterations: Enhanced glycolysis: Elevated expression of GLUT1 and LDHA in alveolar macrophages of smokers, and inflammation exacerbated by accumulation of lactic acid. Lipid accumulation: tar constituents promote CD36-mediated uptake of oxLDL and formation of foam cells (similar to atherosclerotic mechanisms). Smoking exacerbates chronic inflammatory disease by forcing macrophages toward a pro-inflammatory metabolic phenotype via ROS, HIF1α, and other signals. Understanding these mechanisms provides new directions for developing metabolic intervention strategies (Mei et al., 2022).

## 7 Which is the promoter of vascular AS, metabolic reprogramming of immune cells or vascular endothelial dysfunction?

**FIGURE 3:**
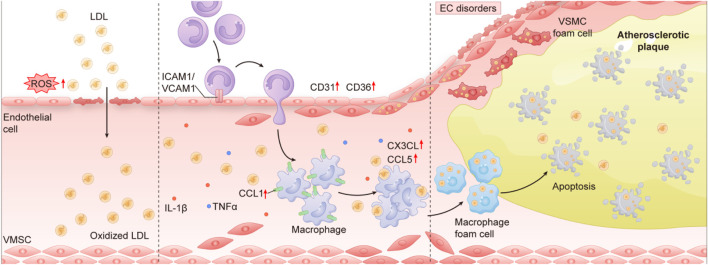
The core pathological chain of “endothelial injury - inflammation - foam cell formation” This picture summarizes the key cellular and molecular mechanisms of occurrence and development, mainly involving endothelial dysfunction, inflammatory response and foam cell formation processes in Atherosclerosis. The first step: Endothelial cell dysfunction occurs first: Monocytes attach to damaged endothelial cells through adhesion molecules such as ICAM1/VCAM1 and migrate to the subcutaneous part of the blood vessels. Part Two: Endothelial cell dysfunction (EC disorders) leads to the release of inflammatory factors (such as IL-1β, TNF-α and CCL2), which further recruit immune cells. Part 3: foam cell Formation and Plaque Development: oxLDL (LDL) is taken up by macrophages, forming macrophage-derived foam cells (Macrophage foam cell). Vascular smooth muscle cells (VSMCS) can also transform into VSMC-derived foam cells, promoting plaque growth (Atherosclerotic plaque). Part Four: Inflammation and Plaque Instability: Persistent inflammatory responses and Apoptosis lead to the expansion of necrotic cores within plaques, increasing the risk of plaque rupture.

In the pathogenesis of AS, vascular endothelial dysfunction isoften considered the initial initiator of disease development, whereas immune cell metabolic reprogramming is a key amplifier driving disease progression and plaque destabilization. Both act synergistically at different stages of AS, but endothelial dysfunction is involved earlier in the pathologic process (As shown in Figure 3). Vascular endothelial dysfunction is the “igniter” of AS. Apolipoprotein B (apoB)-containing lipoproteins (e.g., LDL) pass through the damaged endothelium into the subendothelium, where they undergo oxidative modification (oxLDL) to trigger activation of the endothelium, release of adhesion molecules (VCAM-1, ICAM-1) and chemokines (CCL2), and recruitment of monocytes and T cells. that recruit monocytes and T cells to the lesion site. Oxidative stress and NO imbalance: mitochondrial dysfunction in ECs leads to accumulation of ROS, inhibition of nitric oxide synthase (eNOS), and reduction of vasodilator NO production, triggering endothelium-dependent vasodilatory abnormality and barrier disruption (Lee et al., 2013). Studies have confirmed that increased endothelial permeability and lipid deposition can be observed within weeks of a high-fat diet in ApoE^−/−^mice, predating immune cell infiltration and foam cell formation. INTERVENTION EFFECT: Improvement of endothelial function (e.g., L-arginine supplementation to enhance NO production) significantly delayed early plaque formation.Metabolic reprogramming of immune cells is a “gas pedal” of AS, which amplifies inflammation and plaque progression: monocytes/macrophages and T cells undergo metabolic reprogramming (e.g., enhancement of glycolysis, inhibition of FAO) in the plaque microenvironment to promote the polarization of pro-inflammatory phenotypes (M1 macrophage, Th1/Th17 cells) polarization and release of cytokines such as IL-1β, TNF-α, and IFN-γ, which further activate ECs and recruit more immune cells (Xue et al., 2023). Macrophages form foam cells by over uptake of oxLDL via CD36, while cholesterol efflux is blocked (ABCA1/ABCG1 downregulation), leading to lipid core enlargement and necroinflammation (Fu et al., 2022). Immunometabolic reprogramming plays a dominant role in the transformation of plaques from fatty streaks to complex lesions such as thin fibrous cap plaques. Endothelial dysfunction is the initiating trigger of AS, whereas metabolic reprogramming of immune cells is the central driver of ongoing disease progression. The two are spatially and temporally coupled, but endothelial damage occurs earlier and provides the pathologic basis for subsequent immune responses. Future therapeutic strategies need to balance early endothelial protection with mid- and late-phase immune-metabolic regulation in order to comprehensively interrupt the disease process.

## 8 Does reversal of immune cell metabolic reprogramming improve vascular endothelial dysfunction?

In vascular diseases such as AS, there is a close interaction between immune cell metabolic reprogramming and vascular endothelial dysfunction. Increasing evidence suggests that early reversal of immune cell metabolic reprogramming may ameliorate vascular endothelial dysfunction by attenuating inflammation, oxidative stress, and endothelial injury. Abnormal metabolism of immune cells directly or indirectly damages ECs through multiple pathways: pro-inflammatory factor release: macrophages and T cells secrete more IL-1β, TNF-α, and IFN-γ through the HIF-1α/mTOR pathway in response to enhanced glycolysis (the Warburg effect), activating the endothelial cell NF-κB pathway, inducing adhesion molecule (VCAM-1, ICAM -1) expression and promotes monocyte infiltration (Fan et al., 2022). Oxidative stress diffusion: mitochondrial dysfunction in immune cells leads to excessive release of ROS, which directly damages endothelial cell mitochondria, impairs nitric oxide (NO) bioavailability, and exacerbates endothelium-dependent vasodilatory abnormalities. Lipotoxic delivery: macrophage foaming releases oxidized lipids (e.g., oxLDL, 7-KC), which activate endothelial apoptotic signaling (e.g., caspase-3) and inflammatory vesicles (NLRP3) via the LOX-1 receptor (Wu et al., 2024).

Reversal of immune cell metabolic reprogramming (e.g., inhibition of glycolysis, enhancement of OXPHOS, or cholesterol efflux) can significantly ameliorate vascular endothelial dysfunction by decreasing the release of inflammatory factors and alleviating oxidative stress and lipotoxicity. This strategy provides new therapeutic ideas for diseases such as AS and diabetic vasculopathy, but further optimization of targeting and safety is needed to drive clinical translation. Endothelial function can be improved by targeting key nodes of immune cell metabolism: 1) Inhibition of pro-inflammatory metabolic phenotypes: glycolysis inhibitors: inhibition of macrophage/Th17 cell glycolysis using 2-deoxyglucose (2-DG) or metformin reduces endothelial inflammatory responses by decreasing IL-1β and IL-17 secretion (Pajak et al., 2019). HIF-1α inhibitors: inhibition of HIF-1α (e.g., PX-478) blocks glycolysis-dependent pro-inflammatory macrophage polarization and restores endothelial cell barrier function. Enhancement of anti-inflammatory metabolicpathways: promotion of OXPHOS: PPAR-γ agonists (e.g., pioglitazone) enhance macrophage FAO, promote M2-type polarization, secrete IL-10 and TGF-β, and inhibit endothelial inflammation. Regulation of cholesterol metabolism: LXR agonists (e.g., GW3965) upregulate macrophage ABCA1/ABCG1 expression, enhance cholesterol efflux, and reducefoam cell formation and endothelial lipotoxicity injury (Zheng et al., 2021). Regulating the T cellmetabolic-functional axis: enhancing Treg metabolic adaptation: supplementation with pyruvate or nicotinamide (NAD^+^ precursor) enhances OXPHOS activity in Treg cells and inhibits Th1/Th17 cell-mediated endothelial damage (Mao et al., 2024). Targeting glutamine metabolism: Inhibition of glutaminase (e.g., CB-839) blocks CD8^+^ T cell proliferation and reduces their release of IFN-γ and granzyme B, protecting endothelial integrity (Peyton et al., 2018). Metabolic reprogramming can be reversed by the above strategies. For example, diabetic patients with SGLT2 inhibitors (empagliflozin) showed a decrease in monocyte glycolytic activity and a decrease in circulating endothelial particles, suggesting improved endothelial function.

## 9 Summary

During the onset and progression of AS, immune cell metabolic reprogramming and vascular endothelial dysfunction are two interrelated core mechanisms that together drive inflammatory responses and plaque formation. Immune cells alter energy utilization and modulate their functions (e.g., inflammation, phagocytosis, proliferation) through metabolic reprogramming, which directly affects the AS process (Mouton et al., 2018). Endothelial dysfunction is the initiating link of AS, manifested by increased permeability, inflammatory activation, and vasodilatory abnormalities. The two form a vicious cycle that accelerates AS progression: 1). Immune cell metabolites affect endothelial function: macrophages and T cells release ROS and IL1β that directly damage ECs. Glycolytic intermediates (e.g., lactate) activate endothelial inflammatory pathways through the acidified microenvironment. 2). Endothelial damage drives immunometabolic reprogramming: damaged endothelium secretes chemokines (CCL2, CXCL10) to recruit monocytes and T cells and promote their differentiation toward pro-inflammatory phenotypes. Endothelium-derived ATP deficiency enhances glycolysis-dependent inflammation by activating AMPK and inhibiting macrophage OXPHOS (Yang et al., 2025) 0.3). Cross-over effects of mitochondrial dysfunction: diffusion of endothelial cell mitochondrial ROS to neighboring immune cells exacerbates their oxidative stress and pro-inflammatory metabolic patterns.Release of mtDNA and ATP from immune cells activates endothelial inflammation via TLR9/P2X7 receptors. Endothelial dysfunction provides the “soil” and immune metabolic abnormalities provide the “fuel”, which together lead to the transformation of plaques from a stable to an unstable state. Metabolic reprogramming of immune cells and endothelial dysfunction are the “twin engines” of AS, amplified by a network of metabolic inflammation and oxidative stress (Liu et al., 2025). Future treatments need to target both immune cell metabolic adaptations and endothelial repair mechanisms to break the vicious cycle of inflammation, achieve plaque stabilization or even reversal, and modulate immune metabolism: inhibiting glycolysis (e.g., 2DG, metformin) or enhancing OXPHOS (e.g., PPARγ agonists), However, due to systemic side effects such as hepatic steatosis and heart failure, the treatment of AS with PPARγ agonists remains challengingto promote anti-inflammatory phenotypes (Sandhu et al., 2024). Targeting the HIF1α/mTOR pathway to reduce Th1/Th17 polarization and protect endothelial function: supplementation of eNOS substrate or antioxidants restores endothelial homeostasis. Targeting shear-sensitive genes improves hemodynamic effects, and combined interventions simultaneously modulate immunometabolism and endothelial repair inhibitors with both hypoglycemic and anti-inflammatory effects.
